# Clinical Study on the Implications of Immunological Markers in the Diagnosis of Periodontitis in People with Diabetes Mellitus

**DOI:** 10.3390/dj12060149

**Published:** 2024-05-21

**Authors:** Andreea Dinu, Oana Raluca Antonescu

**Affiliations:** Faculty of Medicine, Lucian Blaga University of Sibiu, 550024 Sibiu, Romania; oanaraluca.antonescu@ulbsibiu.ro

**Keywords:** periodontitis, immunological markers, IL-1β, IL-4, IL-8, TNF-α, diabetes mellitus

## Abstract

The basic idea from which the working hypothesis for this study started is the fact that the only systemic disease today that is clearly linked to periodontal disease by biochemical mechanisms is diabetes mellitus, as well as the clinical finding that diabetes causes a number of specific periodontal changes. Highlighting the biochemical markers of inflammation during periodontal disease in patients diagnosed with type 2 diabetes is the main aim of the study. To achieve this objective, we used the human ELISA kit from Boster Biological Technology Co., Ltd. (Pleasanton, CA, USA), for the detection of IL-1β, IL-4, IL-8 and TNF-α. The data analysis shows that plasma levels of these cytokines are associated with the progression of periodontitis. In conclusion, we can state that the involvement of immunological markers is evident in the pathogenesis of periodontal disease.

## 1. Introduction

The World Health Organization has defined periodontal health as a state free of periodontal inflammation. This allows the individual to function normally and not experience consequences (mental or physical) due to the pathology suffered in the past. A practically applicable definition of periodontal health would be the absence of clinical periodontal inflammation associated with gingivitis or periodontitis (Lang and Bar-told 2018) [1].

Adult periodontitis is a multifactorial disease with a high prevalence in the general population that affects the supporting tissues of the tooth, represented by the periodontal ligaments and the alveolar bone [2]. Periodontitis affects approximately 50% of the adult population worldwide and is considered the most common inflammatory disease; 60% of those affected by periodontitis are elderly. Epidemiological data recently reviewed in the USA show that over 47% of adults are affected, confirming the high prevalence of periodontitis. It is expected that approximately 15% of adults will develop periodontitis in severe forms [3,4]. According to the degree of severity, periodontitis can be staged as follows: stage 1—patients presenting a clinical loss of 1–2 mm of interdental attachment (in the most affected site), and a radiologically visible coronal bone loss 1/3 (maximum 15%), without the absence of teeth due to periodontal reasons; stage 2—patients with a clinical loss 3–4 mm of interdental attachment (in the most affected site), a radiologically visible coronal bone loss 1/3 (15–33%), with no missing teeth due to periodontal reasons; stage 3—patients with a clinical loss of interdental attachment (in the most affected site) greater than 5 mm or with an extension of bone resorption into the middle 1/3 of the root; bone loss is radiologically visible and extended to the middle third, and there are less than four dental units missing due to a periodontal cause; stage 4—patients with a clinical loss of interdental attachment (in the most affected site) greater than 8 mm or with an extension of bone resorption into the apical 1/3 of the root, radiologically visible bone loss extending into the apical 1/3, and more than five dental units missing due to a periodontal cause [5].

The host response in the clinical expression of periodontitis is the key factor, half of which is due to genetic variation and about 20% of which is attributed to bacterial variation, tobacco use, stress and other systemic diseases [6,7,8]. For gingivitis and periodontitis, diabetes is considered a risk factor; this is something highlighted in numerous studies, with clear evidence indicating that an important determinant of this relationship is the level of glycemic control [8,9]. In people with diabetes, the increased prevalence and the greater severity of gingivitis have been highlighted; however, some studies do not show a significant association between diabetes and gingivitis. Most evidence shows that the risk of periodontitis increases in the presence of diabetes. People with diabetes have more severe periodontitis compared to those without diabetes; this was concluded following an in-depth meta-analysis [10,11,12,13,14].

Worldwide, an increase in the incidence of diabetes mellitus has been observed in recent decades. Type 2 diabetes has a growing percentage of people in most countries, with approximately 80% of adults with this condition living in low- and medium-developed countries [15]. In Romania, with an adult population of 14,382,001, there were 1,785,301 cases of diabetes in people aged 20–79 years, and diabetes projections for the years 2030 and 2045 usually show an increasing prevalence of diabetes sugar with age [16]. Diabetes mellitus accentuates the susceptibility and severity of periodontitis, because the impact on the disease process is inversely proportional to the level of glycemic control [17,18,19,20]. The anamnesis and evaluation of diabetes could help to prevent complications of the intervention and improve the follow-up, oral hygiene and oral health of patients [21]. Also, if the infection is severe, the glycemic level is difficult to control and in such a case it may be better to remove the affected teeth and provide oral rehabilitation to the patient [22]. Alimentation and oral rehabilitation helps patients to improve their nutritional health [23].

Obviously, the only systemic condition to which periodontitis is linked through biochemical mechanisms is diabetes; this information is also the basis of the hypothesis of this study. There is a two-way relationship between diabetes and periodontitis, with these both being complex chronic diseases [24]. Adults with diabetes have a three times higher risk of developing periodontitis compared to those without diabetes, with the key to determining the risk being glycemic control [25,26,27]. The effects of diabetes on the host’s inflammatory response have been investigated by multiple studies through highlighting the quantitative or qualitative disturbances of cytokine profiles in adults with periodontitis. The measurement of local inflammatory mediators in the gingival crevicular fluid or the measurement of these inflammatory mediators in the serum, such as interleukin 1 β and tumor necrosis factor-α, in people with diabetes, is supported by researchers, as these inflammatory mediators can lead to the damage of the soft or hard periodontal support [28]. The association of this interleukin in adults with periodontitis and diabetes is highlighted by the most consistent results [29,30,31]. It is important to identify plasma immunological markers in periodontal disease for the early recognition and diagnosis of periodontitis in patients with diabetes, so we can develop prophylactic measures and establish an early therapy to avoid complications. The periodontal disease treatments improve the possibility of the patients being able to manage glycemia and Glycosylated Hemoglobin (HbA1c) [32,33,34].

The aim of our study was to identify the biochemical markers of inflammation during cases of periodontitis in patients diagnosed with type 2 diabetes. Through this study, we aimed to provide further evidence for the literature data.

## 2. Materials and Methods

### 2.1. Subject Recruitment

For the present study, 210 patients presenting to the outpatient dental clinic between 2021 and 2022 were evaluated. From the total number of patients, 66 subjects were included in the research, divided into two homogeneous groups equal in number of subjects, according to the presence or absence of diabetes mellitus. We thus formed a group of 33 subjects with periodontal disease and diabetes mellitus and 33 subjects with periodontal disease but without diabetes mellitus. The selection of patients was made according to the criteria adopted for the classification of periodontal diseases in 2018.

Patients were divided into disease stages according to the severity of periodontal disease as follows:

- Stage 1—patients presenting clinical loss of 1–2 mm of interdental attachment (in the most affected site), radiologically visible bone loss of 1/3 coronal (maximum 15%), without absence of teeth due to periodontal reasons.
- Stage 2—patients with clinical loss of 3–4 mm of interdental attachment (in the most affected site), radiologically visible bone loss of 1/3 coronal (15–33%), no missing teeth due to periodontal reasons.
- Stage 3—patients with clinical loss of interdental attachment (in the most affected site) greater than 5 mm or with extension of bone resorption into the middle 1/3 of the root; bone loss is radiologically visible and extended to the middle third, and there are less than 4 dental units missing due to periodontal cause.
- Stage 4—patients with clinical loss of interdental attachment (in the most affected site) greater than 8 mm or with extension of bone resorption into the apical 1/3 of the root, radiologically visible bone loss extending into the apical 1/3, and more than 5 dental units missing due to periodontal cause.

The inclusion criteria for the study subjects are the following:

- Presence of clinical signs for periodontal disease;
- Gingival retractions;
- Gingival bleeding;
- Halitosis;
- Bone resorption, periodontal pockets;
- Tooth migration or mobility;
- Presence of an OPT X-ray not older than 6 months;
- Age over 25.

The exclusion criteria for the present study are the following:

- Periodontal therapy in the last 12 months,
- Smokers,
- imbalanced cardiovascular disease,
- chronic respiratory or kidney disease,
- osteoporosis or rheumatoid arthritis,
- pregnant or lactating women,
- patients whose data are incomplete or who refuse to participate in the study,
- type 1 diabetes mellitus.

### 2.2. Laboratory Analysis

We aimed to assess the immunological markers IL1β, IL4, IL8 and TNFα, and patients’ plasma was analyzed using enzyme-linked immunosorbent assay (ELISA). We used the human ELISA kit from Boster Biological Technology Co., Ltd. U.S.A. for the detection of IL-1β, IL-4, IL-8, and TNF-α. The ELISA used for the measurements follows the general principles of the method, being based on the sandwich immunoezzymatic assay technique. Patient serums were added to the reaction plate wells, which were previously coated with an antibody, the anti-antigen to be determined. After incubation, during which the antigen and antibody adhering to the plate bind via non-covalent bonds, excess antigen is removed via washing.

After washing, Streptavidin-HRP solution containing peroxidase covalently bound protein, the second antigen detection system, was pipetted over the fixed antigen. Streptavidin binds to biotinylated antibodies and conjugated HRP shows enzymatic activity, with the substrate binding to the active centre of peroxidase. So, the second monoclonal antibody, capable of coupling the antigen, is labelled with an enzyme that will degrade a substrate in the next reaction step. A new washing step follows, after which the chromogenic substrate is pipetted.The degradation of the substrate by the enzyme bound to the second antibody changes the colour of the reaction solution from blue to yellow after addition of the stop solution. The intensity of the colour is proportional to the amount of enzyme, which in turn is proportional to the amount of antigen present in the serum. The optical density reading on the plate was performed at a wavelength of 450 nm.

The washing steps are important in the ELISA technique because they ensure the accuracy of the results. They remove excess initial antigen and then excess antibody bound to the enzyme. Failure to follow the washing steps leads to errors due to the high quantity of enzyme, which will degrade an excessive amount of substrate, thus resulting in false positive reactions.

### 2.3. Statistical Analyses

Data are expressed as frequency and percentages in case of categorical variables and mean (M), standard deviation (SD), and min/max values in case of continuous variables. Comparisons between groups according to IL-1β, IL-4, IL-8, TNFα were carried out using *t*-test. A value *p* < 0.05 was considered statistically significant. All analyses 35 were conducted using SPSS software (Statistical Program for Social Science), version 25.0 [35].

## 3. Results

### 3.1. Patients’ Profile

We conducted a study involving a total of 66 participants, divided equally into two groups (Table 1). Statistical analysis of participants’ origins revealed that 48.5% came from rural areas, while 51.5% came from urban areas, ensuring a balanced and statistically insignificant difference in distribution between groups (Table 2). The gender distribution between subjects with periodontal disease and diabetes and those with periodontal disease alone showed no significant differences, indicating that the variations in the clinical indicator cannot be attributed to gender differences (Table 3). The average age of the participants was approximately 53 years, ranging from 33 to 66 years (Table 4). In terms of age distribution, 42.4% of participants were over 61, while the 41–50 age group comprised 24.2% of the total. The 51–60 and 30–40 age groups each represented close percentages, with 18.2% and 15.2% of subjects, respectively (Table 5).

Regarding the distribution of patients by disease stage, 36.4% were classified as having stage two periodontitis, 30.3% were stage three and 28.8% were diagnosed with stage four periodontitis. Only 4.5% of participants had good oral hygiene and were classified as having stage 1 periodontal disease (Table 6).

### 3.2. Results following the Analysis of Immunological Markers

The statistical analysis of the data reveals a statistical difference in interleukin 1β between the two groups. Thus, in the research group the mean interleukin 1β values are M = 30.09, Sd = 5.68, t = 20.07, df = 64 compared to the control group where M = 9.27, Sd = 1.77, t = 20.07, df = 38.16 (*p* < 0.05) (Table 7). Independent tests show that t = 20.08, df = 64 (Table 8).

We have also noticed a statistical difference between the two groups when analysing the data on interleukin 4 values. The mean value of this interleukin in the research group is M = 35.00 with a standard deviation of Sd = 5.42 (Table 9). Independent tests show that t = 12.04, df = 64 (Table 10). In the control group the data analysis showed the following data M = 23.15, Sd = 1.62, t = 12.03, df = 37.69 (*p* < 0.05).

In the case of interleukin 8 there is a statistical difference between the two groups. Thus, the research group has a mean value of M = 66.00, Sd = 10.19, t = 7.01, df = 64 as compared to the control group where M = 50.79, Sd = 7.15, t = 7.01, df = 57.39 (*p* < 0.05) (Table 11). Independent tests show that t = 7.02, df = 64 (Table 12).

The Analysis of the data on TNFα interleukin values shows a statistical difference between the two groups. Thus, the research group shows M = 54.15, Sd = 7.75, t = 12.01, df = 64 as compared to the control group where M = 32.45, Sd = 6.89, t = 12.01, df = 63.12 (*p* < 0.05) (Table 13). Independent tests show that t = 12.01, df = 64 (Table 14).

### 3.3. Differences between Values by Age Categories

**Figure 1:**
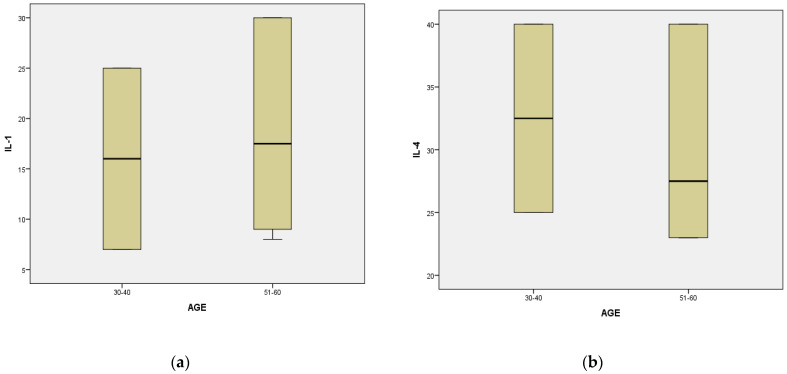

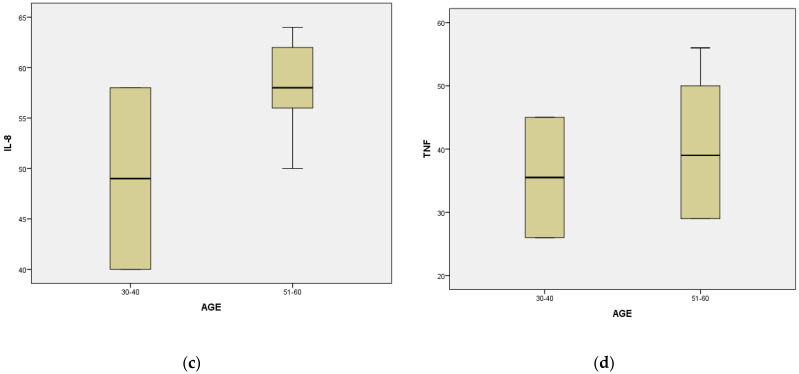
Graphic representation of the mean differences between the age categories: (**a**) Differences between the age categories 30 and 40 and 51 and 60 regarding IL-1 (*p* > 0.05), (**b**) Differences between the age categories 30 and 40 and 51 and 60 regarding IL-4 (*p* > 0.05), (**c**) Differences between the age categories 30 and 40 and 51 and 60 regarding IL-8 (*p* < 0.05), (**d**) Differences between the age categories 30 and 40 and 51 and 60 regarding TNFα (*p* > 0.05).

Analysis of immunological marker data for age groups 30–40 and 51–60 showed statistical changes only in the level of interleukin 8 where for the age category of 30–40 years we have M = 49.00, Sd = 9.48, t = −2.86, df = 20 (*p* < 0.05) in comparison with the age category 51–60 years where M = 58.00, Sd = 4.89, t = −2.71, df = 12.92 (*p* < 0.05) (Figure 1). For the immunological markers interleukin 1β, interleukin 4 and TNFα no statistical changes were found (*p* > 0.05) (Table 15).

### 3.4. Differences According to the Stage of Disease

The analysis of data between stages two and three reveals highly statistically significant differences for interleukin 1β in stage two M = 10.46, Sd = 5.75, t = −6.67, df = 42 compared to stage three periodontal disease where M = 21.80, Sd = 7.50, t = −5.53, df = 35.19. Likewise, regarding interleukin 4 it is observed that M = 25.88, Sd = 5.53, t = 4.07, df = 42 in stage two and M = 34.19, Sd = 7.83, t = −3.94, df = 33 in stage three (*p* < 0.05) (Table 16).

There were also highly statistically significant differences in interleukin 8 and TNFα levels between the two stages of periodontal disease. For interleukin 8 in stage two M= 49.42, Sd = 7.33, t = 5.48, df = 42, and in stage three M = 58.90, Sd = 2.67, t = −5.88, df = 30.00. For TNFα in stage two M = 30.63, Sd = 6.23, t = −7.60, df = 42, and for stage three M = 45.75, Sd = 6.95, t = −7.53, df = 38.65 (*p* < 0.05) (Table 17).

Analysis of immunological markers reveals statistically significant differences between the two stages of periodontal disease analysed, respectively stage two and stage four for all variables except interleukin 4 (*p* > 0.05) (Table 18).

For interleukin 1β at stage two we observe that M = 10.46, Sd = 5.75, t = −6.97, df = 41, compared to stage four where M = 29.21, Sd = 11.49, t = −6.49, df = 25.07. For interleukin 8 in stage two disease, we have M = 49.21, Sd = 7.33, t = −7.11, df = 41, compared to stage four where M = 70.21, Sd = 11.73, t = −6.75, df = 28.69, and for TNF α in stage two periodontal disease we observed that M = 30.63, Sd = 6.23, t = −11.94, df = 41, while in stage four M = 57.47, Sd = 8.51, t = −11.51, df = 32.00 (*p* < 0.05) (Table 19).

**Figure 2:**
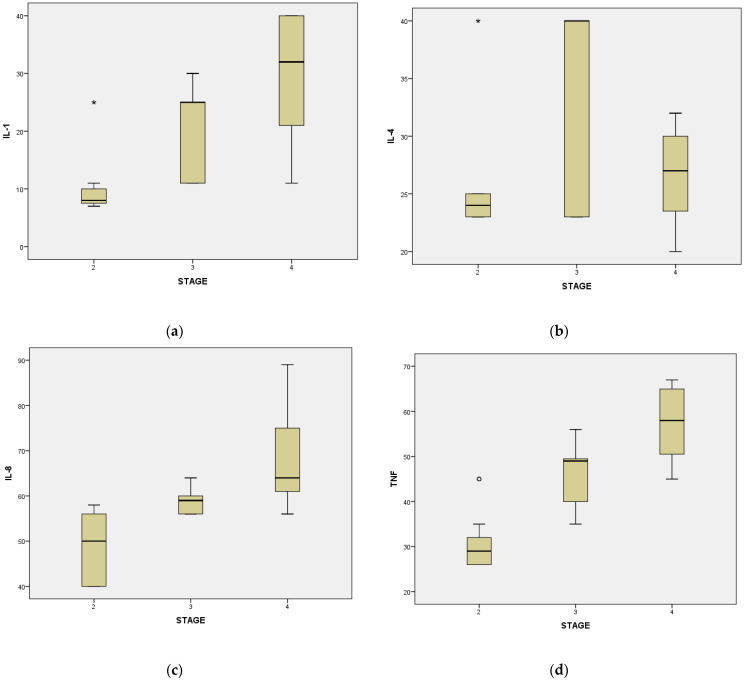
Graphical representation for cytokine values according to periodontal disease stages: (**a**) Differences relating to IL-1, (**b**) Differences relating to IL-4, (**c**) Differences relating to IL-8, (**d**) Differences relating to TNFα. (*p* < 0.05).

Cytokine values according to the stages of periodontal disease are represented in Figure 2.

## 4. Discussion

Several studies suggest a bidirectional relationship between diabetes and periodontitis: advanced periodontal diseases can negatively affect the metabolic control of diabetes and can cause the level of glycosylated hemoglobin to rise in people without this pathology. In addition, periodontal disease, by its mere presence, seems to be a risk factor for the development of systemic complications of diabetes [36,37,38]. It has been shown that the subgingival microflora can enter the systemic circulation through the well-vascularized ulcerated epithelium of the periodontium and can even have a direct effect in promoting systemic inflammation and insulin resistance. The resolution of the inflammatory process in the periodontal tissues as a result of the performed therapy causes the reduction in the inflammatory mediators at the local level, and therefore the values of these markers in the circulation [39,40]. Increased levels of inflammatory cells and mediators in the systemic circulation are associated with periodontal disease, which increases insulin resistance, general health indicators and glycemic control can be improved by periodontal treatment in adults with diabetes [41,42,43]. It is very important to intercept the “active phase” of periodontitis and to identify the initial periodontal lesions when they are not yet evident. Specific biomarkers are released in the early stages of the immune response, and to achieve these results, periodontists worldwide need high-tech tools to highlight these biomarkers [44]. Periodontal tissue is clinically healthy when there is a balance between host and microbes; an important immune response occurs when the deposition of bacterial plaque occurs, many inflammatory substances are released in the periodontal tissues that would be eligible as biomarkers for the early diagnosis of periodontitis. Diagnosing periodontitis in the early stages without becoming clinically visible, is the goal of a “futuristic” periodontal diagnosis in the near future, thus avoiding the progression of the disease. The local microbiota and the host’s immune response are the most important etiological and risk factors related to the initiation and progression of periodontitis [45,46,47]. The role of cytokines is extremely important in the progression of periodontitis. Homeostasis, the delicate balance within the body to maintain stable internal conditions, plays a crucial role alongside inflammatory processes in the initial response against pathogens and stimuli. These processes occur primarily at barrier sites and involve various types of connective tissue cells interacting with lymphocytes and accessory cells. Central to these interactions are cytokines, which serve as pivotal regulators. Research indicates that interleukin-1β, a pro-inflammatory cytokine, plays a significant role in exacerbating periodontal damage. Specifically, it actively stimulates osteoclastic activity, contributing to bone resorption associated with periodontal disease. Moreover, interleukin-1β’s angiogenic properties promote the formation of blood vessels in inflamed tissues, further exacerbating the inflammatory response. This underscores the multifaceted role of cytokines, particularly interleukin-1β, in orchestrating immune responses and tissue damage in inflammatory conditions like periodontal disease [30,48]. The study by Kenneth S. et al. demonstrates a strong predictor of susceptibility to severe periodontitis in adults are specific genetic markers associated with increased levels of interleukin 1, and studies by Kimbel et al. provide strong data that IL-1 activity is involved in the contribution of osteoporosis associated with endocrine changes [45,49].

Data from the literature show increased concentrations of 1β in the serum of patients with periodontal disease and diabetes mellitus. Elevated levels of IL-1β in diabetes activate the expression of other inflammatory cytokines and amplify the pro-inflammatory milieu, temporarily increase insulin secretion, which may be detrimental to metabolism [50,51]. Our results confirm that the serum level of interleukin 1β in subjects with periodontitis and diabetes has a much higher mean compared to subjects without diabetes, this explains the more advanced degree of bone damage in the research group and reinforces the literature data.

The health of the periodontal tissues is closely related to interleukin 4, which has an important and central role in the pathogenesis of periodontitis, this being demonstrated in numerous studies in the specialized literature [52]. Changes in its levels were evident in the evolution of periodontitis by studying this cytokine at the plasma level. In the study by CIRELLI T. et al., patients with periodontitis were compared with healthy ones and no changes were obtained in the average level of this cytokine [53]. In our current study, we noted significant statistical differences between the two groups when examining the levels of this particular cytokine. Our findings revealed a notable increase in the average cytokine levels among periodontal patients compared to the control group. In our study, there is a statistically significant difference between the mean IL8 plasma levels between the research group and the control group, the subjects with diabetes and periodontitis have the mean values of this cytokine much increased compared to the control group. It has been found at the human level that glucose produces oxidative stress that causes an increase in the amount of interleukin 8 in the gingival epithelial cells [54]. The excessive inflammatory response of the host with diabetes in periodontal disease, according to Kashiwagi et al., is based on the hypothesis of a possible involvement of epithelial cells [55]. Fokema et al., as well as other authors in the literature, have demonstrated that there are elevated plasma levels of interleukin 8 in subjects with periodontitis. In specialized investigations, evidence indicates that while there exists a correlation between periodontitis and elevated levels of this cytokine in crevicular fluid, the impact of the disease on plasma interleukin levels was found to be diminished [56,57]. Interleukin 8 plays an important role in the pathogenesis of diabetes and periodontal disease, its increased circulating levels being associated, in the presence of periodontitis, with diabetes. Circulating concentrations of interleukin 8 are not influenced by individual interleukin 8 genetic variants or the presence of individual periodontal bacteria. Our research unveiled a rise in the levels of this interleukin as individuals age. Aging is known to induce structural and functional alterations in the immune system, heightening vulnerability to chronic illnesses. This phenomenon elucidates the higher incidence of periodontitis observed in older individuals. However, aging cannot cause periodontal disease unless there is concomitant periodontal inflammation. Microbial aggression through prolonged exposure has an increased effect on the onset and severity of periodontitis in older ages. It is predicted that periodontal disease will become more common among the elderly in the future [58]. The molecular basis of our findings needs to be addressed further, as they may have some diagnostic implications.

The tumor necrosis factor plays a very diverse role in the body, being represented by numerous cytokines organized in the form of a large family, these cytokines have numerous ligands and receptors. An essential role in the response to infection injuries is played by tumor necrosis factor-α, which is a pro-inflammatory cytokine produced by most macrophages, it also plays a role in angiogenesis, apoptosis and other physiological processes. High serum levels of this cytokine have been shown in adults with periodontitis [59]. This TNF has also been shown to have a direct impact on diabetes by playing an important role in the development of insulin resistance [60]. Multiple polymorphisms have been identified in the promoter region of TNF-α, and the 308G/A and 863C/A polymorphisms may contribute to susceptibility to periodontitis according to a meta-analysis [61]. Madureira et al. demonstrate that TNF levels were increased in both gingival crevicular fluid and serum of periodontitis patients. The elevation in tumor necrosis factor (TNF) levels could be linked to its role in bone metabolism. Research suggests that TNF plays a role in regulating the expression of RANKL in gingival epithelial cells. Moreover, TNF has been observed to induce apoptosis in gingival fibroblasts and epithelial cells, while concurrently suppressing the production of extracellular matrix in gingival fibroblasts. This multifaceted involvement of TNF underscores its significant impact on periodontal health and bone homeostasis. These findings suggest that TNF may contribute to periodontitis by compromising the oral mucosal barrier. In addition, it is important to highlight the fact that the levels of circulating TNF, which is involved in the pathogenesis of various systemic diseases, can link periodontitis to conditions such as diabetes and rheumatoid arthritis, thus exacerbating systemic inflammatory processes. [24,62]. Our research found increased levels of TNFα in the serum of individuals affected by both periodontitis and diabetes. This discovery underscores the significant role of tumor necrosis factor-alpha (TNF-α) as a proinflammatory cytokine in the resorption of alveolar bone and damage to periodontal tissues. The correlation between periodontal disease and elevated levels of TNF-α suggests that it serves as a key mediator in the local bone degradation observed in this condition. Research indicates a notable increase in plasma TNF-α levels among individuals with periodontal disease, which aligns with the loss of gingival attachment and deepening of periodontal pockets. These findings strongly imply a direct link between TNF-α levels and the progression of periodontal disease [63,64,65,66,67,68].

In our current investigation, we assessed the levels of immunological markers associated with different stages of the disease. Our findings indicated a notable rise in interleukin 1β, 8, and TNFα levels during stages two, three, and four of the disease. This signifies a consistent elevation in the average levels of these cytokines as periodontal disease progresses. Furthermore, our results uncovered a correlation between disease advancement and age, with an increase in disease stage coinciding with worsening conditions as individuals age. At present, diabetes stands out as the sole systemic condition clearly associated with periodontal disease through biochemical pathways. The hyperglycemic state tends to be exacerbated by acute infections and inflammatory conditions, leading to heightened insulin secretion and its augmented utilization in peripheral tissues. This intricate interplay complicates the metabolic management of diabetes [58]. The correlation between diabetes and periodontitis is very little studied in the specialized literature, most studies include control groups of healthy patients, our study having an extra originality by including the control group of patients with diabetes and periodontitis. It was reported by Marconcini et al. that salivary oxidation and a certain oxidative level in blood can help in the assessment of diabetes and periodontal inflammation, because oxidation and inflammation are linked [69]. Further research could strengthen the evidence base so that a range of markers can be established to assess periodontitis so that morbidity and risk of surgery can be prevented. By carrying out this study we aimed to complete the data from the literature.

This study has some limitations related to the size of the study population or the fact that the temporal relationship between cytokine release and disease development is not explored in detail. The study also notes age-related changes in cytokine levels, but does not fully control for potential confounding factors associated with aging, such as comorbidities or changes in oral hygiene habits over time. The 2018 classification of periodontitis, as proposed by the World Workshop, identified smoking as a significant factor that contributes to the advancement of periodontitis. Patients with periodontal disease who smoke typically experience more severe symptoms and face a greater risk of disease progression compared to non-smokers [70]. Another limitation of this study is that we did not include smoking subjects in the current research and that we did not delineate diabetes patients according to the level of hyperglycemic control, which would probably have required additional groups of participants for such a study, these aspects will be addressed in our future studies.

## 5. Conclusions

There is an association between periodontitis progression and plasma levels of inflammatory markers studied.

Our findings indicate that as periodontitis progresses, there is a notable increase in cytokines 1β, 8 and tumor necrosis factor-α. This increase contributes to the development of a systemic proinflammatory condition.

We have shown that ageing leads to continued progression.

We have achieved the aim of the study to identify plasma immunological markers in periodontitis, which will help to quickly establish the diagnosis and initiate early treatment in order to avoid complications and increase the quality of life of patients with type 2 diabetes and periodontitis.

## Figures and Tables

**Table 1:** Patient group description.

	Frequency	Percent	Valid Percent	Cumulative Percent
Witness group	33	50.0	50.0	50.0
Research group	33	50.0	50.0	100.0
Total	66	100.0	100.0	

**Table 2:** Distribution of subjects according to origin.

	Frequency	Percent	Valid Percent	Cumulative Percent
Rural	32	48.5	48.5	48.5
Urban	34	51.5	51.5	100.0
Total	66	100.0	100.0	

**Table 3:** Distribution of subjects according to gender.

	Frequency	Percent	Valid Percent	Cumulative Percent
Female	33	50.0	50.0	50.0
Male	33	50.0	50.0	100.0
Total	66	100.0	100.0	

**Table 4:** Distribution of subjects according to age.

	N	Minimum	Maximum	Mean	Std. Deviation
Subjects’ age	66	33	66	53.39	10.156
Valid N (listwise)	66				

**Table 5:** Distribution of subjects on age categories.

Age Categories	Frequency	Percent	Valid Percent	Cumulative Percent
61+	28	42.4	42.4	42.4
51–60 41–50	12 16	18.2 24.2	18.2 24.2	60.6 84.8
30–40	10	15.2	15.2	100.0
Total	66	100.0	100.0	

**Table 6:** Distribution of subjects according to disease stage.

Disease Stage	Frequency	Percent	Valid Percent	Cumulative Percent
Stage four	19	28.8	28.8	28.8
Stage three	20	30.3	30.3	59.1
Stage two	24	36.4	36.4	95.5
Stage one	3	4.5	4.5	100.0
Total	66	100.0	100.0	

**Table 7:** Means and standard deviations for Interleukin 1β.

Inflammatory Marker	Group of Subjects	N	Mean	Std. Deviation	Std. ErrorMean
Interleukin 1β	Research group	33	30.09	5.686	0.990
	Witness group	33	9.27	1.773	0.309

**Table 8:** Tests between independent samples for Interleukin 1β.

Inflammatory Marker	Levene’s Test for Equality of Variances		*t*-Test for Equality of Means			
	F	Sig.	t	df	Sig. (2-Tailed)	Mean Difference
Interleukin 1β	29.069	0.000	20.078	64.000	0.000	20.818
			20.078	38.161	0.000	20.818

**Table 9:** Means and standard deviations for Interleukin 4.

	Group of Subjects	N	Mean	Std. Deviation	Std. ErrorMean
IL 4	Research group	33	35.00	5.420	0.943
	Witness group	33	23.15	1.623	0.282

**Table 10:** Tests between independent samples for Interleukin 4.

Inflammatory Marker	Levene’s Test for Equality of Variances		*t*-Test for Equality of Means			
	F	Sig.	t	df	Sig. (2-Tailed)	Mean Difference
IL 4	160.279	0.000	12.031	64.000	0.000	11.848
			12.031	37.690	0.000	11.848

**Table 11:** Means and standard deviations for Interleukine 8.

Inflammatory Marker	Group of Subjects	N	Mean	Std. Deviation	Std. ErrorMean
IL8	Research group	33	66.00	10.192	1.774
	Witness group	33	50.79	7.158	1.246

**Table 12:** Tests between independent samples for Interleukine8.

Inflammatory Marker	Levene’s Test for Equality of Variances		*t*-Test for Equality of Means			
	F	Sig.	t	df	Sig. (2-Tailed)	Mean Difference
IL8	2215	0.142	7.017	64.000	0.000	15.212
			7.017	57.390	0.000	15.212

**Table 13:** Means and standard deviations for TNFα.

	Group of Subjects	N	Mean	Std. Deviation	Std. ErrorMean
TNFα	Research group	33	54.15	7.759	1.351
	Witness group	33	32.45	6.892	1.200

**Table 14:** Tests between independent samples for TNFα.

Inflammatory Marker	Levene’s Test for Equality of Variances		*t*-Test for Equality of Means			
	F	Sig.	t	df	Sig. (2-Tailed)	Mean Difference
TNFα	1.388	0.243	12.010	64.000	0.000	21.697
			12.010	63.124	0.000	21.697

**Table 15:** Differences between the age categories 30 and 40 and 51 and 60 regarding immunological markers.

Inflammatory Markers	Levene’s Test for Equality of Variances		*t*-Test for Equality of Means			
	F	Sig.	t	df	Sig. (2-Tailed)	Mean Difference
Interleukin 1β (valori)	1.233	0.280	−0.627	20.000	0.537	−2.667
			−0.632	19.751	0.535	−2.667
IL 4	0.142	0.710	0.685	20.000	0.501	2.333
			0.686	19.361	0.501	2.333
IL8	37.879	0.000	−2.868	20.000	0.010	−9.000
			−2.714	12.922	0.018	−9.000
TNFα	7.585	0.012	−1.023	20.000	0.318	−4.917
			−1.042	20.000	0.310	−4.917

**Table 16:** Differences between the means of variables in stage two and stage three of periodontal disease.

Inflammatory Markers	Stage of Disease	N	Mean	Std. Deviation	Std. ErrorMean
Interleukin 1β (values)	Stage two	24	10.46	5.756	1.175
	Stage three	20	21.80	7.509	1.679
IL 4	Stage two	24	25.88	5.535	1.130
	Stage three	20	34.10	7.833	1.752
IL8	Stage two	24	49.42	7.330	1.496
	Stage three	20	58.90	2.673	0.598
TNFα	Stage two	24	30.63	6.233	1.272
	Stage three	20	45.75	6.950	1.554

**Table 17:** Analysis of the mean difference for immunological markers between stage two and stage three periodontal disease.

Inflammatory Markers	Levene’s Test for Equality of Variances		*t*-Test for Equality of Means			
	F	Sig.	t	df	Sig. (2-Tailed)	Mean Difference
Interleukina 1β (values)	5.265	0.027	−5.670	42.000	0.000	−11.342
			−5.535	35.192	0.000	−11.342
IL 4	10.148	0.003	−4.071	42.000	0.000	−8.225
			−3.946	33.333	0.000	−8.225
IL8	24.191	0.000	−5.481	42.000	0.000	−9.483
			−5.886	30.003	0.000	−9.483
TNFα	0.581	0.450	−7.607	42.000	0.000	−15.125
			−7.531	38.659	0.000	−15.125

**Table 18:** Differences between the means of variables in stage two and stage four of periodontal disease.

Inflammatory Markers	Stage of Disease	N	Mean	Std. Deviation	Std. ErrorMean
Interleukin 1β (values)	Stage two	24	10.46	5.756	1.175
	Stage four	19	29.21	11.497	2.638
IL 4	Stage two	24	25.88	5.535	1.130
	Stage four	19	26.89	4.557	1.045
IL8	Stage two	24	49.42	7.330	1.496
	Stage four	19	70.21	11.736	2.692
TNFα	Stage two	24	30.63	6.233	1.272
	Stage four	19	57.47	8.514	1.953

**Table 19:** Analysis of the mean difference for immunological markers between stage two and stage four periodontal disease.

Inflammatory Markers	Levene’s Test for Equality of Variances		*t*-Test for Equality of Means			
	F	Sig.	t	df	Sig. (2-Tailed)	Mean Difference
Interleukin 1β (values)	12.184	0.001	−6.977	41.00	0.000	−18.752
			−6.495	25.079	0.000	−18.752
IL 4	0.008	0.930	−0.647	41.00	0.521	−1.020
			−0.662	40.918	0.511	−1.020
IL8	6.325	0.016	−7.114	41.00	0.000	−20.794
			−6.751	28.693	0.000	−20.794
TNFα	2.865	0.098	−11.940	41.00	0.000	−26.849
			−11.518	32.007	0.000	−26.849

## Data Availability

The data presented in this study are available upon request from the corresponding author because they are part of a larger study.
